# To Readers of "The Hospital."

**Published:** 1921-08-13

**Authors:** 


### To Readers of "The Hospital."

A nurse inquires if any one will exchange their copy of The Hospital for her copy of *The Nursing Mirror*, each to be posted on Tuesdays. We shall be glad to forward any offers, which should be addressed "Exchange," c/o the Editor, The Hospital, 28 and 29 Southampton Street, Strand, London, W.C. 2.

